# Trends of Cardiovascular Disease Mortality in Relation to Population Aging in Greece (1956 - 2015)

**DOI:** 10.2174/1874192401812010071

**Published:** 2018-07-31

**Authors:** Natasa Kollia, Alexandra Tragaki, Aristomenis I. Syngelakis, Demosthenes Panagiotakos

**Affiliations:** 1 Department of Nutrition and Dietetics, School of Health Science and Education, Harokopio University, Athens, Greece; 2 Department of Geography, School of Environment, Geography and Applied Economics, Harokopio University, Athens, Greece; 3 School of Social Sciences, Hellenic Open University, Patras, Greece; 4 Faculty of Health, University of Canberra, Bruce, Australia; 5 School of Allied Health, College of Science, Health and Engineering, LA TROBE University, Melbourne, Australia; 6 Department of Kinesiology & Health RUTGERS, School of Arts & Life Sciences, The State University of New Jersey, New Brunswick, NJ, USA

**Keywords:** Population aging, Cardiovascular disease, Mortality, Life expectancy, Fertility, Demographic changes

## Abstract

**Background:**

Demographic dynamics and decreasing trends in mortality from chronic diseases are major contributors to the phenomenon of population aging. The purpose of the present study was to examine the association between cardiovascular disease (CVD) mortality and demographic indicators, in Greece the past 60 years.

**Methods:**

Life Expectancy at birth (LE), population age structure, fertility rates (TFR) and all-cause, CVD mortality rates were retrieved (data provided by the Hellenic Statistical Authority, 1956-2015). In order to test the research hypothesis time-series analysis was conducted.

**Results:**

Increasing trends in LE and in the older age (>65 or >80 years) groups’ share and declining trends in TFR were recorded. CVD mortality, after an upward course, showed decreasing trends during 1988–2009, accounting for the 96% and 97% increment in LE in men and women respectively. However, newer records (2010-2015) show a new upward trend. The declining trends in TFR were highly associated with the shifts towards the upper part of the population age pyramid.

**Conclusion:**

Population aging is a historically unprecedented event that cannot be avoided, deterred or alleviated. Its negative effects act cumulatively with the recent increases in cardiovascular mortality, especially in the light of the ongoing economic crisis which is expected to further exacerbate the existing contrasts. A possible way to successfully cope with the new demographic realities is to unlock an, up till now largely overlooked, opportunity named “healthy aging”.

## 1 INTRODUCTION

Recent trends in cardiovascular disease (CVD) mortality show remarkable declining rates in European countries and North America [1]. Despite this evidence, CVD is still the number one cause of death in most OECD (Organisation for Economic Co-operation and Development) countries, accounting for more than one-third of all deaths in 2015 [2]. Furthermore, the prevalence of common CVD risk factors like hypertension, obesity, hypercholesterolemia and diabetes have increased [3, 4], especially in younger people [1], implying that cardiometabolic co-morbidity does not necessarily follow mortality trends. Furthermore, since cardiovascular health seems to be highly associated with socioeconomic factors^5^ and the reduction in CVD mortality over the past years has not been felt equally in all sectors of society - the most impressive improvements in cardiovascular health benefited the richest societal groups and countries [5] - the investigation of the most current CVD mortality trends (*i.e.* under the context of the ongoing economic crisis) is of particular value.

Alongside, during the last decades, substantial demographic changes have been recorded. Globally, life expectancy at birth has increased by approximately 25 years since the 1950s [6], probably due to the declining mortality rates attributed mainly to the spectacular improvements in health care, in pharmacological and other treatment and in general to the advances in medical science. The increment in life expectancy, while undeniably a major achievement, coupled with falling fertility rates, reshapes population pyramids and shifts volumes towards older age groups, a phenomenon widely known as population aging. Europe is by all means the oldest region of the planet. Currently, half of the European population is over 42 years of age, while 1 out of 4 Europeans is above 60 years of age [7].

Of all areas, health care is particularly sensitive to a population's age-structure. Increasing shares of elder and old age groups are expected to affect: (i) the demand, (ii) the organization and delivery, and, (iii) the cost of health care systems. In parallel, in the context of the economic crisis, the increased cost of health care for the elderly creates the risk of raising ethical issues concerning rationing in Medicine (the allocation of scarce resources) [8]. Moreover, as longevity allows greater number of persons to reach higher ages, previously uncommon conditions become frequent, if not dominant morbidity causes. Particularly, CVD risk levels increase with age since the key risk factors are either directly age-related - like hypertension, diabetes, elevated cholesterol levels - or have a cumulative harmful effect, like tobacco use, obesity and physical inactivity [9]. Against this challenging demographic background and taking into account that age is one of the most dominant determinants of cardiovascular health, CVDs are expected to remain the leading cause of death in the years to come.

In the context of the aforementioned considerations, this paper aims to examine the effect of the trends in CVD mortality on longevity and on population age structure in Greece, during 1956 – 2015 and to discuss the implications and challenges arising from this favorable, but, also complex phenomenon involving the modern western world.

## 2 METHODS

### 2.1 Official Statistics

Demographic indicators, such as life expectancy at birth (LE), fertility rates (TFR), population age structure (1960-2015) and all-cause, CVD mortality rates (1956-2015) have been calculated using data provided by the Hellenic Statistical Authority (EL.STAT.) [10] and the EUROSTAT (Directorate-General of the European Commission) Population Projections-Baseline scenario [10-13].

### 2.2 Statistical Analysis

**Fig. (1):**
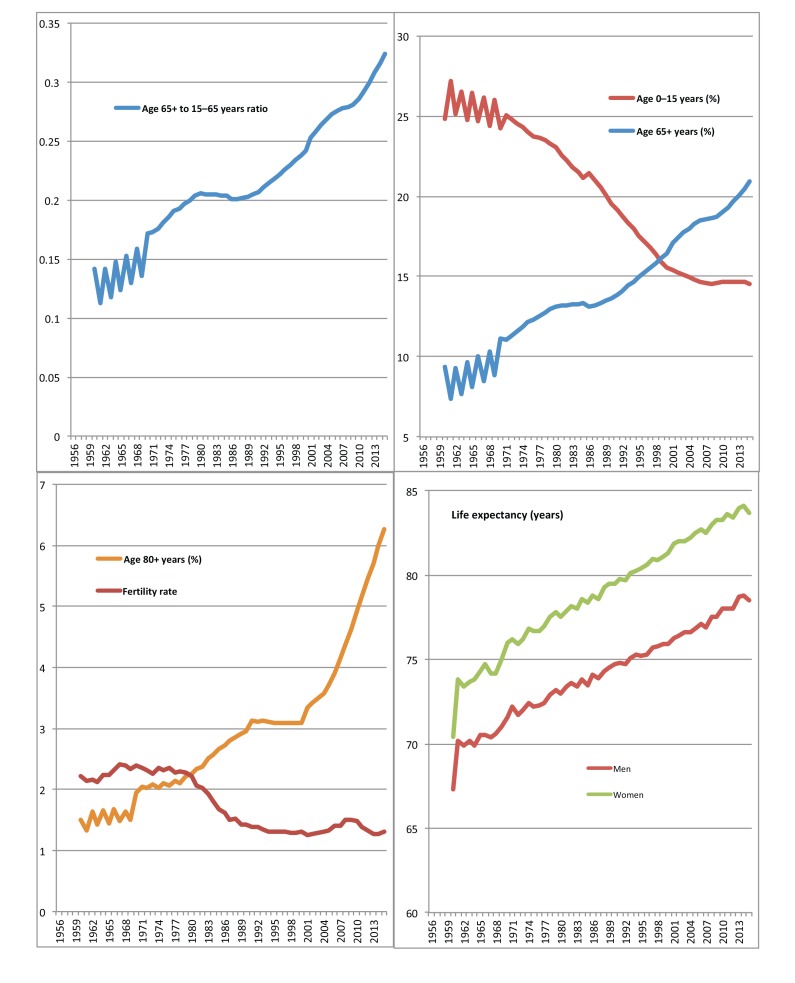
Demographic time trends in Greece (1956–2015); source: Hellenic Statistics Authority [10].

**Fig. (2):**
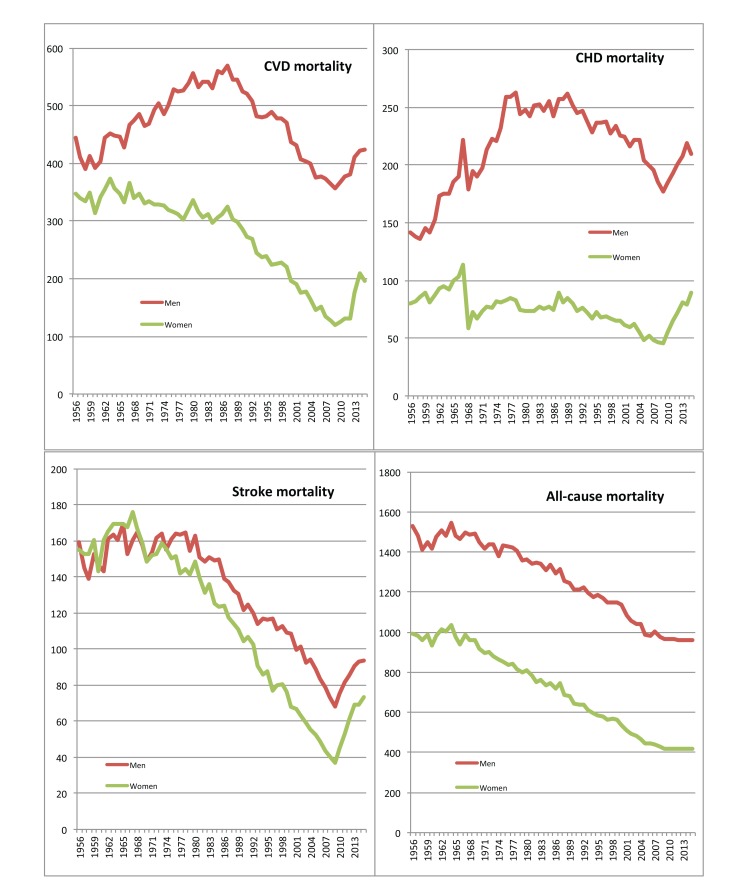
Mortality time trends in Greece (1956–2015); source: Hellenic Statistics Authority. *CVD: cardiovascular disease; CHD: coronary heart disease. Mortality rates were calculated per 100,000 persons* [10].

Mortality rates were presented per 100,000 persons. Time trends in the demographic indices and in mortality rates were graphically presented (Figs. **1** and **2** respectively). Time-series analysis was conducted to evaluate the effect of all-cause and CVD mortality and fertility trends during 1956-2015 (independent determinants) on life expectancy and on the age structure of the Greek population (outcomes), in men and women separately. Due to the presence of a significant interaction between time and the independent variables, a stratified by year analysis was implemented. The estimated b-coefficients along with the 95% confidence intervals, the corresponding *p*-values and the adjusted *R^2^* were provided. All reported *p*-values were based on two-sided tests and overall statistical significance level was set at 5%. STATA software, version 14 (MP & Associates, Sparta, Greece) was used for all statistical analyses.

## 3 RESULTS

### 3.1 Population Aging in Greece

According to the latest official statistics (population estimates 2015), the median age of the Greek population has increased by approximately 10 years since 1970s (*i.e.* from 32.3 to 43.4 years), and it is expected to further increase in the future (Table **1**). According to the projections provided by EUROSTAT (baseline scenario) [11] by 2030, half of the Greek population will be over 50 years age; more than one-third will be above 65 years and almost 9% will be above 80 years, in contrast to the youngest population segment which will be limited to just 11% (Table **1**). In particular, the group aged 0-14 comprise a rapidly shrinking part of the Greek population; already <20% in the 1990s, their share dropped down to as low as 14.5% in 2015. At the other end of the age spectrum, those above 65 years old currently represent 27% of total population, which is 31% higher than in 2000. The growth rate of those above 80 years old is even more spectacular, as their share more than doubled since the 1990s (Table **1**). The time trends of the Greek population age structure during the last 60 years are presented in Fig. (**1**).

### 3.2 Life Expectancy and fertility Trends

The time trends of life expectancy at birth are similar for both men and women and exhibit a linearly increasing course with men having consistently, approximately 3 years shorter estimates (Fig. **1**). In 2015, life expectancy was increased by 13 years for women and by 11 years for men compared with 1960 with an increasing rate of 0.20 [95% confidence intervals (CIs): 0.20-0.21), *p*<0.001] and 0.17 (95% CIs: 0.16-0.17, *p*<0.001) per year, respectively. Using the National Vital Statistics for the years 2000-2015 as provided by EL.STAT [10], the highest life expectancy gain was observed for the 60 to 79 years old men and women, followed by the over 80 years old people (data not shown).

In contrast, the fertility rate in Greece has substantially decreased during the past decades. After a relatively constant course until the late 1970s, a downward trend followed (Fig. **1**) with a declining rate of 0.2 units per decade (*p*<0.001).

### 3.3 Mortality Trends

Until the late 1980s, CVD and coronary heart disease (CHD) mortality rates were following an increasing course (especially for men) (Fig. **2**). The peak of CVD death rate for men was in 1987 (570 deaths per 100,000 persons) (*i.e.*, 257 CHD deaths per 100,000 persons, 137 stroke deaths plus other forms of cardiovascular diseases). CVD and CHD death rates for women were essentially stable until the early or mid-80s. From 1988 to 2009, this course was reversed with an annual decreasing rate of 9 deaths for both genders (95% CIs: 8.3-10 for men and 8.0-9.2 for women). A new upward course of 13 and 18 deaths annually (per 100,000 persons) for men and women respectively (*p*<0.001) was recorded during the most recent years (2010-2015). In both genders CHD mortality time trends in Greece over the last 60 years followed the same course as CVD. Stroke deaths were relatively stable until 1970s, followed by a considerable decline until 2009 and a sharp increase afterwards (2010-2015) for both men and women (Fig. **2**). The time trends for all-cause mortality rate differed, since a stable decreasing course was observed from the 1950s and onwards for both genders (Fig. **2**), with an annual decrement of 11 deaths per 100,000 persons for men (95% CIs: 10-11, *p*<0.001) and 12 deaths for women (95% CIs: 11-12, *p*<0.001).

### 3.4 Effects of Mortality and Fertility Time trends on Life Expectancy and Population Aging

Time series analysis showed that, for men, CVD and CHD mortality rates were positively associated with life expectancy at birth until 1987. During 1988-2009 this association was reversed with the reduction in CVD mortality accounting for the 96% in life expectancy rise for men and 97% for women (Table **2**). The decrement in all-cause mortality rate since the 1950s was associated with an annual increase in life expectancy by approximately 0.15 years (*p*<0.001) for both genders and with a substantial growth in the relative representation of the older age-groups compared with the younger ones (Table **2**). Furthermore, from the early 1970s and onwards, the decrement in fertility rate accounted for the 79% of the 0-15 years age-group shrinkage and for the 43% of the increase in the ratio 65+ to 15-65 years (*p*<0.001 in both cases).

## 4 DISCUSSION

Greece is an aged country with already half of its population >43 years of age while population projections suggest that the aging process will continue in the decades to come, due to fertility and mortality dynamics which favour the increasing of older groups to the detriment of younger ones, but also lead to the reduction of death rates from diseases such as CVD, especially in the older age-groups [1]. Furthermore, the continued prolongation of the life span observed over the last decades, acts cumulatively and aggravates the phenomenon of the aging population.

The Greek population has shown a rapid increase of the elderly people percentage while its median age is currently among the highest in Europe [12]. In 2015, the share of Greeks that had celebrated their 80^th^ birthday (6.3%) was the second highest in EU, very close behind Italy (6.4%) [12]. Simultaneously, life expectancy increased by almost 4 years since the 1990s (although slower than other countries) and by almost 10 years since the 1970s, a figure that gives Greece a World Life Expectancy ranking of 25 among the WHO countries [13]. This phenomenon does not only characterize the Greek population. The vast majority of modern economies are aging; some of them at a fast pace and exhibit lowering fertility rates and increases in life expectancy [14, 15]. The combined effect of increased longevity and low fertility is responsible for a great part of the population “greying”.

Although Greece was among the last countries to join Europe’s declining fertility course, these late though sharp, falling fertility rates soon reached the lowest levels world-wide. In particular, compared with 1.4 in the 1990s and to 2.20 in the 1980s, in 2015 fertility rate was only 1.3 births/woman, that is far below the value of 2 - which is considered as the replacement rate for a population resulting in relative stability - and is indicative that population is decreasing in size and growing older [16]. According to EUROSTAT [11] estimates, by 2030, the overall size of the Greek population is projected to be smaller, but much older. Although projections are nothing more than projections, based on assumptions, it is currently beyond any doubt that the greying of the Greek population will impose serious financial and social burdens [17].

*Prima facie*, CVD mortality trends herald optimistic news as death rates have been reduced quite substantially in the last 4 decades, although differentiations have been observed according to the county-region, the statistical method applied and the source of mortality information [18, 19]. In many European countries, in 2009 death rates from heart disease were <50% as compared with the 1980s [1]. Similar trends have been recorded for stroke and CHD in particular, as expected since they are both a part of the general category of CVD. These declines in CVD mortality have been mainly attributed to the progress made in cardiovascular care and to efficient cardiovascular treatment (*e.g.* use of statins and better antihypertensive medication) [1], a breakthrough in medical science that was experienced and became strongly perceptible in Greece from the late ‘80s and onwards [20]. Unfortunately, a second and more insightful look raises some justifiable concerns: i) the most recent evidence indicates a renewed increase in CVD mortality rates which seems strongly affected by the undergoing economic crisis, ii) CVD remains the leading cause of death in Greece, responsible for approximately 5 in every 10 deaths (*i.e.* 48%; 26% for CHD and 22% for stroke) during 2000-2015, whereas 90% of those deaths concerned adults above 60 years old [10, 13]. Similarly, 32% of the deaths globally were attributable to CVD, making it the leading cause of death worldwide [21], iii) the demographic transition is a major driver of CVD; the same time that death rates have fallen globally by 22%, the raw numbers of CVD deaths have increased by 41% due to the aging and growth of the world’s population [21], iv) substantial differences have been observed between High-Income (HIC) and Low- and Middle-Income Countries (LMIC); from 1990 to 2013, in HICs a 43% decline in CVD death rates has been seen while no significant changes have been observed in the CVD-related number of deaths [21]. On the other hand and during the same period, in LMICs, the decrease in CVD mortality rates was much lower (*i.e.* only 13%) while being accompanied by a simultaneous increase of the raw numbers in CVD deaths by 66% [21], v) during the last decades, evidence of an increase in diabetes and obesity prevalence among adults has been reported while in some regions similar increases have been observed for smoking, physical inactivity, dyslipidaemia, hypertension and psychological distress (*i.e.* CVD risk factors) [1, 3, 4, 22-24], vi) it has also been formulated that the current national trends in CVD mortality have masked local increases, especially among younger adults (*i.e.* below 65 years old) [25], vii) it has been hypothesized that the recorded declining CVD mortality rates are mainly related to the older ages, while the corresponding trends among younger adults seem to be plateauing or even rising as the increase in the prevalence of obesity and diabetes is cancelling the gains from reducing smoking [1] while, as far as premature deaths are concerned, CVD is enhanced steadily, beginning at ages as young as 30-34 years, where it accounted for 11% of all deaths [20], and, viii) notably in Greece, it has been suggested that the recent declining trends in all-cause mortality [26] and in CVD death rate particularly, do not necessarily go hand in hand with corresponding morbidity reductions. This is supported by several facts such as: the increasing environmental pollution that enhances the propagation of adverse pathophysiological processes, linked with atherosclerosis, including genetic, haemodynamic, metabolic, oxidative and inflammation parameters [27]; the observed deviation from the healthy and cardioprotective Mediterranean dietary pattern during the past decades [28]; the increment in the key CVD risk factors that has been discussed earlier; and the phenomenon of population aging and social upheavals clearly suggest that shifts towards the upper part of the age pyramid entail a reshuffling in the disease prevalence [21].

Population aging is a historically unprecedented event that cannot be avoided, deterred or alleviated. Much of this demographic phenomenon is attributed to the progress made towards longevity, which is a great achievement. The same applies to the spectacular progress made in medical and pharmacological science with consequence the substantial decrease in mortality rates. Spectacular though this progress may seem, it is not free from negative side effects. In order to fully exploit these undoubtedly positive steps, for the benefit of society by promoting well-being the side effects arisen must be efficiently and constructively answered, as longer lifespan does not necessarily mean healthier additional years. Life expectancy at 65 is 19 years (for both sexes), while healthy life expectancy is only 8 years. Stated differently, <42% of the remaining years of a 65-year old Greek are expected to be in good health [29, 30]. An option is to timely prepare societies for the years to come through the restructuring of the health care system, the review of the budgetary constraints and the implementation of innovative ideas. A possible way to successfully cope with the new demographic realities is to unlock an, up till now largely overlooked, opportunity named “healthy aging” which is described by the World Health Organization as a process of developing and maintaining the functional ability that enables well-being in older age [31]. Functional ability is made up of the intrinsic individual’s ability, the environmental characteristics and the interactions between them while well-being theoretically involves the concepts of satisfaction, fulfilment and happiness [31]. Longevity in good health seems today the “comparative advantage” of Europe “*vis à vis*” all other aging societies [32]. Europeans in their sixties and early seventies are more physically robust, more mentally alert and better educated than ever before [33] but efforts in this direction should be continued and stepped up so that healthy aging can be achieved for the entire population regardless of societal, economic, racial, cultural or religious characteristics. The results of the study can be also interpreted using the classic Omran’s theory of epidemiological transition [34]. Factors involved in the epidemiological transition are demographic changes, biological factors (microorganisms), environmental factors, social, cultural and behavioural factors and the practices of modern medicine [35]. The dominant role of SDH (Social Determinants of Health) in population health status has been widely recognized [36]. In addition, the particular impact of the SDH on CVD, in view of the epidemiological transition, has been documented [37] and thus, due to the recent financial crisis in Greece, further study of the impact of the social conditions on the CVD in the Greek population during the last decade could be considered.

*Strengths and limitations*: although the present work has documented valuable official data describing mortality and demographic trends and the association between them over a 60-year period, unfortunately no data was available on CVD morbidity or on CVD raw death numbers during the same period.

## CONCLUSION

In light of the projected growing proportion (and number) of old aged population and under the assumption of no major changes in either, risk factors or treatment, CVD prevalence is expected to increase in the decades to come in Greece. The burden of CVD and its cardiometabolic disorders is high, particularly in the urban Greek population, despite the various strategic plans and public health actions developed in the past years. The aforementioned findings underlie the need for emerging and focused prevention strategies in order to reduce the CVD burden at population level, especially in view of the dramatic population aging and the current economic crisis.

## Figures and Tables

**Table 1:** Population age distribution for the Greek population, during the years 1970–2030.

**Year**	**1970**	**1980**	**1990**	**2000**	**2015**	**2030^a^**
**Total Population, N**	8,300,399	8,780,514	9,584,184	10,120,892	10,775,627	9,944,658
*<15 years old (%)*	24.2	23.1	19.5	14.7	14.5	11.7
*65 – 80 years old (%)*	11.1	13.1	13.7	17.3	20.9	27.2
*>80 years old (%)*	2.0	2.3	3.0	3.5	6.3	8.7
**Median age** (years)	32	34	36	38	43	50

^a^ EUROSTAT Population Projections according to baseline scenario [11].

**Table 2:** Time-series analysis results that evaluate the effects of mortality and fertility (1956–2015) (independent variables) on life expectancy and population ageing (dependent variables).

–	**Gender**	**Time**	**Life Expectancy** **(in years)**			**Population Age Distribution**					
						**65+ years / 15–65 years rate**			**Percent of 0–15 years**		
b (95% CI)	p	R^2^	b (95% CI)	p	R^2^	b (95% CI)	p	R^2^
**CVD^a^ Mortality** (per 10 deaths/100.00 people)	Men	1956–1987	0.31 (0.26, 0.36)	<0.001	0.85	0.01 (0.004, 0.01)	<0.001	0.76	-0.29 (-0.37, -0.21)	<0.001	0.68
		1988–2009	-0.16 (-0.17, -0.14)	<0.001	0.96	-0.01 (-0.005, -0.004)	<0.001	0.97	0.31 (0.26, 0.36)	<0.001	0.89
		2010–2015	0.14 (0.05, 0.22)	0.011	0.79	0.01 (0.004, 0.01)	0.001	0.92	-0.01 (-0.04, 0.02)	0.420	0.17
	Women	1956–1987	-0.68 (-1.0, -0.35)	<0.001	0.39	-0.01 (-0.02, -0.01)	<0.001	0.64	0.67 (0.44, 0.89)	<0.001	0.57
		1988–2009	-0.22 (-0.24, -0.20)	<0.001	0.97	-0.01 (-0.01, -0.005)	<0.001	0.97	0.34 (0.29, 0.39)	<0.001	0.92
		2010–2015	0.07 (0.01, 0.13)	0.036	0.64	0.004 (0.001, 0.01)	0.009	0.82	-0.01 (-0.02, 0.01)	0.459	0.14
**All-cause Mortality** (per 10 deaths/100.00 people)	Men	1956–2015	-0.14 (-0.15, -0.13)	<0.001	0.92	-0.003 (-0.003, -0.002)	<0.001	0.90	0.22 (0.21, 0.23)	<0.001	0.96
	Women	1956–2015	-0.16 (-0.17, -0.15)	<0.001	0.95	-0.003 (-0.003, -0.002)	<0.001	0.91	0.21 (0.20, 0.22)	<0.001	0.97
**Fertility Rate** (per 1 unit/woman)	All	1956–1970	3.7 (0.25, 7.2)	0.039	0.36	0.11 (0.01, 0.21)	0.041	0.32	-4.6 (-11, 1.8)	0.141	0.14
		1971–2015	-4.6 (-5.5, -3.7)	<0.001	0.69	-0.07 (-0.09, -0.04)	<0.001	0.43	8.2 (6.9, 9.5)	<0.001	0.79

^a^ CVD: cardiovascular disease. Regression b-coefficients illustrate the effect of CVD or all-cause mortality, and fertility rate (independent variables) on the following outcomes: population life expectancy, the rate of (65+) / (15-65) years old people, and the % of people between 0 – 15 years. For example, among men during 1956-1987, an increase of 10 CVD deaths/100,000 people was associated with: 0.31 years increase in Life Expectancy, 0.01 increase in the rate (65+) / (15-65) years old people and a decrease of 0.15 of the % of people between 0-15 years old.
